# Factors affecting branch failures in open-grown trees during a snowstorm in Massachusetts, USA

**DOI:** 10.1186/2193-1801-3-720

**Published:** 2014-12-10

**Authors:** Brian Kane, John T Finn

**Affiliations:** Department of Environmental Conservation, 160 Holdsworth Way, Amherst, MA 01003 USA

**Keywords:** Branch failure, Open-grown tree, Weakly attached branch, Branch morphology

## Abstract

In October 2011, a snowstorm in the northeastern USA caused many branch failures of many tree species commonly planted in urbanized settings. Immediately following the storm, we assessed 1,764 trees for possible snow-induced damage and factors affecting it on the campus of the University of Massachusetts in Amherst, MA, USA. Nearly all failures were of branches, most of which were not defective. We used logistic regression to assess whether the probability of branch failure differed among species, diameter at breast height (DBH) and the presence of a defect or leaves increased for different species. We also measured branch morphology of (i) branches that did and did not fail for one angiosperm species and (ii) all branches on a sub-sample (stratified by DBH) of three individuals of seven other angiosperm species. Probability of branch failure differed among species. It also increased with greater DBH in eight of ten species studied, decreased when defects were present in four of ten species, and increased in one species when leaves were present. The relationship between branch failure and DBH appeared to be due to the correlation between DBH and branch morphology, which was mostly similar among species. As DBH increased, so did the mean diameter and length of primary branches, and the cumulative diameter of secondary branches. In contrast, branch slenderness decreased with increasing DBH. Combined, these factors presumably expedited the accumulation of snow on branches due to greater surface area and less flexibility. This explained why most failed branches were not defective. Since the frequency of intense storms is predicted to increase with global climate change, urban foresters should consider the timing of leaf senescence when selecting deciduous trees, to reduce the likelihood of failure of open-grown, deciduous trees in urbanized areas.

**Electronic supplementary material:**

The online version of this article (doi:10.1186/2193-1801-3-720) contains supplementary material, which is available to authorized users.

## Introduction

The heavy accumulation of ice and snow on tree stems and crowns can cause large economic losses to forests (Nykänen et al. 1997; Valinger and Fridman 1997; Heigh et al. 2003). In urbanized areas, ice- and snow-induced tree failure can damage infrastructure and injure people. In the snowstorm described herein, damage was severe: the United States Federal Emergency Management Agency (FEMA) disbursed over $71.2 M in public assistance grants in Massachusetts alone (Anonymous, 2012). Stem failures due to the accumulation of snow occur mainly in coniferous trees in cold climates associated with longer presence of snow cover (Nykänen et al. 1997). In addition to heavy snow loading, ice loading also induces tree failure (Van Dyke 1999; Irland 2000; Smith 2000; Bragg et al. 2003; Kraemer and Nyland 2010). The accumulation of ice or snow on branches is mainly controlled by meteorological conditions such as temperature, precipitation and wind speed, and less by branch morphology (Jones 1998; Satterlund and Haupt 1970; Nykänen et al. 1997; Schmidt and Gluns 1991). The occurrence of ice- and snow-induced damage of trees can be well predicted by (i) the amount of accumulated ice or snow and (ii) tree size (Nykänen et al. 1997; Päätalo et al. 1999; Proulx and Greene 2001).

Few studies have considered ice- and snow-induced damage to open-grown trees in urbanized settings, which are often in close proximity to the built infrastructure (Hauer et al. 1993; Sisinni et al. 1995; Rhoades and Stipes 2007). In the absence of neighbors, open-grown trees typically assume a decurrent form that is often altered by management practices such as pruning. For open-grown trees, assessing parameters like stem taper, which is commonly associated with different modes of failure (Nykänen et al. 1997; van Dyke 1999; Zhu et al. 2006), is difficult. Contradictory findings on the resistance of open-grown trees to ice loading exist (Hauer et al. 1993; Sisinni et al. 1995; Rhoades and Stipes 2007). For obvious reasons, in urbanized settings, damaged trees are usually cleaned up quickly, which hinders data collection, although Nock et al.’s (2013) method may overcome this.

In urbanized settings, it is important to gauge the effects of defects on the likelihood of failure, in addition to meteorological and species-specific factors. Practitioners commonly assess the risk of failure of an individual tree by examining its structural integrity. Evaluating the severity of some defects, however, is still based largely on experience rather than empirical data. For example, there are very few studies that have quantified the breaking strength of stems of large, open-grown trees with and without defects (Kane and Clouston 2008; Kane 2014). In urbanized settings, tree failure can damage property and injure persons, which, in the United States, are sometimes associated with costly litigation (Mortimer and Kane 2004).

To help practitioners improve resilience of the urban forest in anticipation of future storms, the objective of this study was to determine whether attributes that are commonly included in a tree inventory (species, DBH and defects such as decay and weakly attached branches) or affect the likelihood of failure were associated with the modeled probability of failure of trees that we observed. Attributes that affect the likelihood of failure include those that affect both the accumulation of snow or ice (branch morphology, crown architecture, and presence of leaves) and the resistance of branches to failure [branch morphology and wood modulus of rupture (MOR)]. We hypothesized that branch morphology and the presence of leaves on branches facilitated the accumulation of snow, inducing stress that exceeded branch strength.

## Methods

### Assessment of failure and tree attributes

On October 29–30 2011, up to 75 cm of snow accumulated across western parts of Massachusetts, USA (USDA Hardiness Zone 5). On the campus of the University of Massachusetts in Amherst, MA (42.4 North, 72.5 West, 45–122 m elevation), 17 cm of snow accumulated during the storm, in which temperatures hovered around 0 C, and wind speed averaged less than 2.9 m/s. These conditions led to the accumulation of heavy, wet snow on tree crowns. At 2120 h, the weather station on the campus stopped collecting data due to a power failure. However, the weather station at Westover Air Force Base in nearby Chicopee, MA recorded maximum wind gusts up to 9.6 m/s for the storm. Such speeds are not expected dislodge accumulated snow from trees (Nykänen et al. 1997).

In November 2011, within three weeks of the storm, we surveyed 1,764 trees on the campus. We chose areas to survey by randomly assigning numbers to an alphabetical list of buildings, parking lots, and roads on campus. We measured all trees that were (i) within 5 m of the infrastructure and (ii) at least 5 cm DBH. We recorded the following attributes for each tree: its location (name of nearest aspect of infrastructure), genus and species, DBH, whether and what part (trunk, root, branch) of the tree failed, whether and what type of a defect was associated with the failure, whether and what type of a defect in the tree had not failed, and whether the tree was in leaf.

Time constraints necessitated that we chose attributes that multiple observers could record quickly and accurately. We considered trees to have leaves if greater than 50% of the crown was in leaf. We considered only the following defects, which practitioners commonly assess (Smiley et al. 2011): weakly attached branches (“V”-shaped attachment, co-dominant stem, presence of included bark), decay, cracks, leans, and girdling or damaged roots. We only recorded observable decay, rather than using indicators such as fruiting bodies or carpenter ants. Because multiple observers examined trees, we did not quantify the severity of defects to avoid observer bias. To keep the tree as the unit of observation in the analyses, we did not analyze trees with multiple failures. Since multiple observers collected data, the lead author reviewed photos of all assessed trees to ensure accuracy of individual observations.

Several months after the storm, broken or hanging branches remained in some trees. To investigate whether aspects of crown architecture contributed to the likelihood of failure, we climbed nine pin oaks (*Quercus palustris* Muenchh.) that had at least one broken or hanging branch and measured the following attributes: DBH; height; and the height, diameter, length, azimuth, and attachment angle of all primary branches greater than 5 cm in diameter. We also measured the diameter and length of every secondary and tertiary branch greater than 5 cm in diameter. Leaf surface area increases non-linearly with branch diameter (Cummings 1941; Rothacher et al. 1954; Weiskittel et al. 2009), but pruning of open-grown trees may confound that relationship. Summing the diameter of higher-order branches presumably reflected the total leaf surface area borne by a primary branch more accurately than primary branch diameter because branches less than 5 cm in diameter are not often pruned. From diameter (d) and length (l), we calculated branch slenderness (l/d), which influences the deflection and stress distribution along the length of a branch. The height, angle, and azimuth of primary branches may have also affected the accumulation of snow—Nock et al. (2013) showed that ice preferentially accreted higher in crowns, and branch attachment angle is often associated with the formation of included bark, which can weaken the attachment (Smiley 2003). For branches that failed, we noted whether a defect was associated with the failure.

A preliminary analysis revealed that DBH was related to the likelihood of failure for many species. To investigate why this occurred, in 2013, we randomly selected three individuals of seven other angiosperm species, stratified by DBH, to ensure that one individual was near the first, fifth, and ninth deciles of the range of DBH for the species. After measuring DBH, we climbed trees to measure tree height and the following attributes of primary branches greater than 3 cm in diameter: height, diameter, length, azimuth, and attachment angle. We also measured the diameter of every secondary branch greater than 3 cm in diameter to estimate total leaf surface area borne by primary branches. It was not possible to measure the length of secondary branches accurately because of safety concerns associated with climbing.

### Data analyses

We did not analyze 41 trunk (stem) and root failures of the trunk or roots and 2 dead trees, focusing only on branch failures. We conducted separate analyses for the three data sets we collected: (i) tree failures observed after the snowstorm, (ii) intensively-measured branches of pin oaks, and (iii) intensively-measured branches of seven angiosperm species.

For trees observed immediately following the storm, we used the Pivot Table function in Excel© (Microsoft Corp., 2011), to conduct a frequency analysis to find the most commonly observed species. To ensure sufficient observations in logistic regression models (described in the next paragraph), we selected species with at least 48 individual observations and a range of DBH.

For each species, we developed logistic regression models to predict failure based on the following explanatory variables: DBH, leaf (0 = leafless, 1 = in-leaf), defect (described below), and their interactions. For trees that failed and a defect was associated with the failure, 1 (“yes”) was entered; if no defect was associated with the failure, 0 (“no”) was entered. For trees that did not fail, if a defect were present, 1 was entered; if no defect were present, 0 was entered. For each species, we used the following steps to select an appropriate model. First, we created coplots, a conventional approach to illustrating logistic regression models, that included the number of trees that failed and did not fail for each DBH within the combinations of leaf and defect. For some species (see the Results), we did not include the effect of leaf because of insufficient individuals with or without leaves. Secondly, we developed binomial generalized linear models (GLM) to investigate the effect of explanatory variables and their interactions on the probability of failure. We also centered the intercept in the model on DBH so that it reflected the probability of failure for a tree of mean DBH of a particular species. Thirdly, we selected the best GLM using likelihood ratio tests (the “drop1” command in R, Zuur et al. 2009) and compared it to the null model. Fourth, we checked coplots for evidence of non-linearities; for species where they were apparent, we developed a generalized additive model (GAM). A smoother for DBH was used to predict the probability of failure for each combination of defect (yes or no) and leaf (in-leaf or leafless), and linear terms were also included for main effects and interactions.

We selected final models using the Akaike Information Criterion (AIC). For most species (Table 1), we used GLMs instead of GAMs—even though the GAM had a smaller AIC than the GLM—because the GAM was not significant (p < 0.01). Since most species (except red maple and white pine) had a relatively small sample size, for each combination of defect and, where applicable, leaf, we adopted more conservative alpha levels for logistic models. We attempted to account for tree location (i.e. revealing different site conditions such as soils, exposure, elevation, etc.), by adding it as a random effect in the generalized linear mixed effects model (GLMM). We compared the best GLMM to the best GLM using AIC, but the GLMM did not add any insight to the GLM for any species.

**Table 1:** **For each species, the number (n) of observations; mean DBH (cm) followed by the standard deviation in parentheses; percentage of trees that failed, had leaves, and had a defect; probability of failure [p(f)] of a tree of mean DBH, followed by the standard error in parentheses; and whether p(f) was significantly different from a 50% chance of failure**

Species	n	DBH	Fail	Leaf	Defect	p(f)^z^	p
Red maple	185	27 (15)	31%	51%	51%	0.27 (0.41)	0.012
Green ash	79	27 (10)	24%	6%	42%	0.11 (0.73)	0.005
Honeylocust	102	34 (14)	35%	7%	24%	0.35 (0.26)	0.013
Red pine	62	33 (9)	5%	100%	23%	0.00 (2.85)	0.015
White pine	156	38 (16)	28%	100%	33%	0.01 (0.87)	<0.001
London planetree	50	40 (17)	76%	96%	14%	0.78 (0.38)	0.001
Pin oak	102	43 (21)	54%	96%	30%	0.70 (0.32)	0.007
Red oak	63	53 (25)	72%	83%	22%	0.84 (0.39)	<0.001
Littleleaf linden	55	44 (13)	69%	60%	22%	0.59 (0.53)	0.497
Liberty elm	54	26 (8)	28%	30%	63%	0.62 (0.75)	0.511

^z^Calculated from the centered intercept in the logistic regression model.

Excluding pin oak branches that failed at a defect, we used an analysis of variance (ANOVA) that included the random effect of tree to determine whether any branch attributes differed between branches that failed and did not fail. For branches of the other seven angiosperm species, we examined scatter plots to determine whether attributes of primary branches were correlated with DBH. For attributes that were not correlated with DBH, we used ANOVA to determine whether they varied among species, including the random effect of tree in the model. For attributes that appeared to be correlated with DBH, we used an analysis of covariance (ANCOVA) to determine whether they varied among species, including DBH as a covariate. Since branch length and diameter influence the total leaf area borne by a primary branch as well as its angle of deflection, both of which presumably influence the amount of snow that accumulates on a branch, we also used ANCOVA to investigate differences in the cumulative diameter of secondary branches between eight angiosperm species including primary branch slenderness as the covariate. We used ordinary least squares (OLS) regression to determine if wood modulus of rupture (Kretschmann 2010) was associated with the probability of failure among species. We used R (http://www.r-project.org) to conduct the logistic regressions and PROC MIXED, PROC REG, and PROC GLM (SAS Institute, Cary, NC) to conduct the ANOVAs, OLS regression, and ANCOVAs.

## Results

### Observed failures and tree attributes

**Figure 1:**
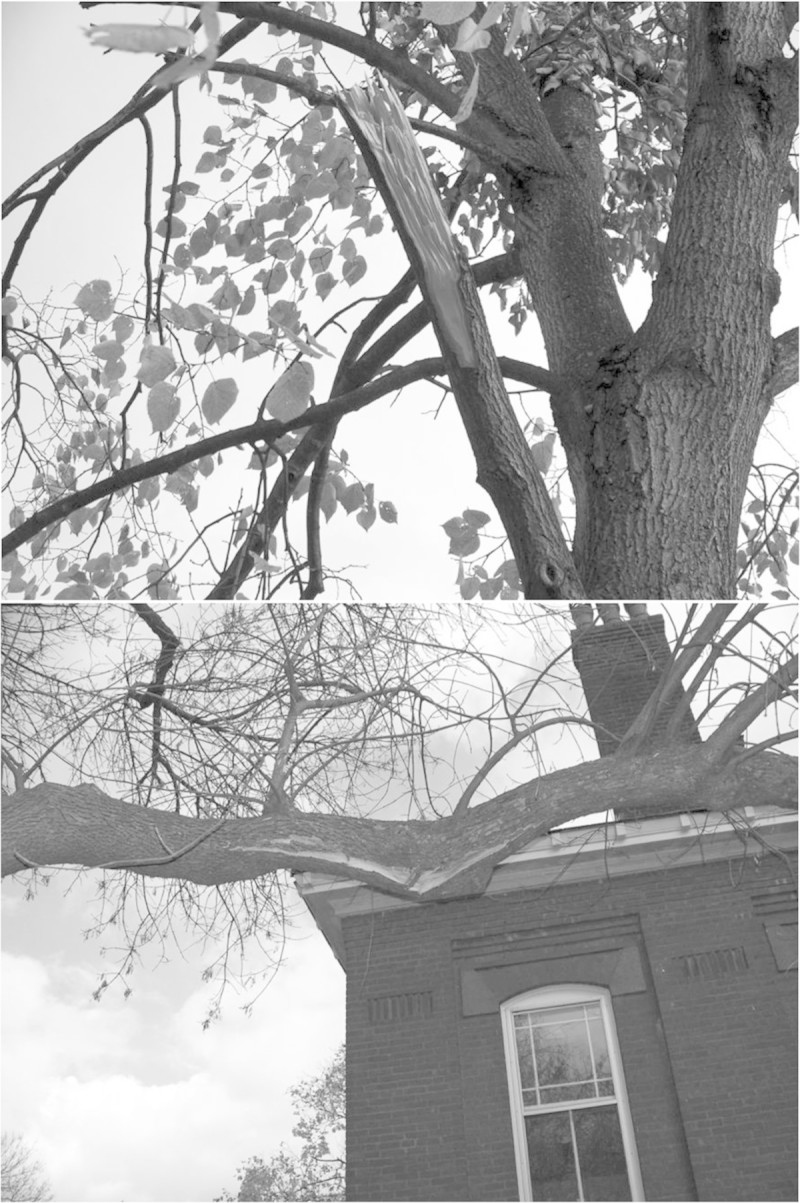
**Bending (top) and shear failures of branches of Littleleaf linden and Green ash, respectively.**

Most branch failures appeared to be in bending or shear (Figure 1). Of 510 failures, 22% were associated with a defect. However, 91% of all assessed trees had at least one defective branch. Nearly all failures associated with a defect had a weakly attached branch (80%), decay (12%), or a combination of those defects (4%). Of observed defects that did not result in branch failure, most were weakly attached branches (78%), decay (13%), and a combination of these defects (2%). Individuals of 128 species were observed, of which 10 had at least 48 observations (Table 1). Wide ranges existed among the ten species with respect to DBH, and the percentage of trees that failed, had leaves or a defect (Table 1). For 5 species, more than 80% of individuals were in-leaf and for 2 species fewer than 10% of individuals were in-leaf. For all but two species, fewer than half of individuals had a defect (Table 1).

Probability of failure varied among species (Table 1), but was not correlated with MOR (r^2^ = 0.23, p = 0.228). It was significantly greater than 50% for London planetree (*Platanus* x *acerifolia* (Air.) Willd.), pin oak, and red oak (*Quercus rubra* L.); and significantly less than 50% for green ash (*Fraxinus pennsylvanica* Marsh.), white pine (*Pinus strobus* L.), and, marginally, for honeylocust (*Gleditsia triacanthos* L.), red maple (*Acer rubrum* L.), and red pine (*Pinus resinosa* Ait.).

**Figure 2:**
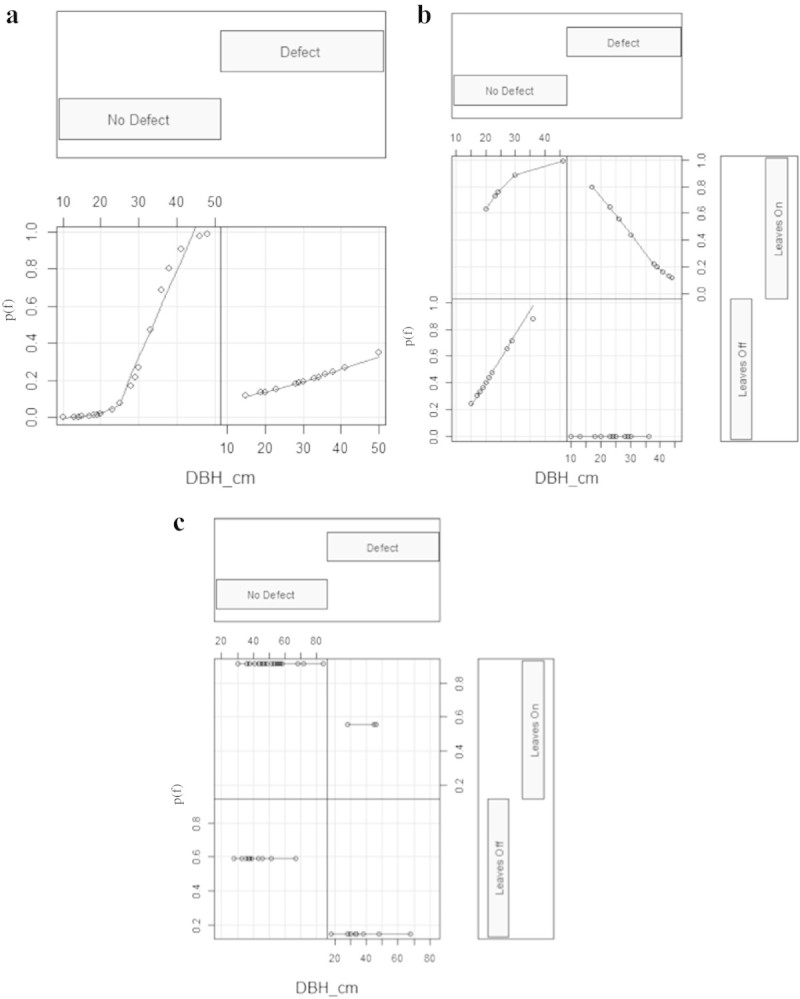
**Coplot of expected probability of failure [p(f), on the ordinate] with respect to DBH (on the abscissa) of a) green ash, b) Liberty elm and c) littleleaf linden.** Coplots are divided in halves (for green ash) or quarters (for Liberty elm and littleleaf linden) which correspond to presence or absence of defects, leaves, or their combination. DBH is indicated on the abscissa for each half or quarter of each coplot. Within each half or quarter of the coplot for each species, circles represent individual trees and lines represent the smoother created by the best generalized linear model. Coefficients for significant explanatory variables are included in Table 2.

Figure 2 shows the probability of failure for the range of DBH within each combination of the main effects of defect and leaf (where applicable) for three species (see also Table 2). The following species had too few trees with (green ash, honeylocust) or without leaves (red pine, white pine, London planetree, pin oak, red oak) to include the effect of leaf as an explanatory variable in the logistic models.

**Table 2:** **Explanatory variables in logistic regressions that were significant (p < 0.01) or marginally significant (p < 0.05) with respect to probability of failure for each species**

Species	Effect	Logit	z	p(>|z|)
Red maple	Defect	−1.58 (0.43)	−3.65	<0.001
	No Defect*DBH	0.15 (0.05)	3.15	0.002
Green ash	DBH	0.30 (0.10)	2.88	0.004
	No Defect*DBH	0.26 (0.12)	2.22	0.026
Honeylocust	DBH	0.04 (0.02)	2.11	0.035
Red pine	DBH	0.21 (0.09)	2.25	0.024
White pine	DBH	0.09 (0.02)	4.17	<0.001
London planetree	DBH	0.05 (0.02)	2.18	0.029
Pin oak	DBH	0.07 (0.02)	4.45	<0.001
	Defect	−1.34 (0.61)	−2.21	0.027
Red oak	Defect	−2.33 (0.67)	−3.47	0.001
Littleleaf linden	Defect	−2.11 (0.84)	−2.50	0.013
	Leaf	1.97 (0.74)	2.65	0.008
Liberty elm	No Defect*DBH	0.27 (0.14)	1.97	0.049

Logit of the odds ratio for each effect are followed by the standard error in parentheses.

‘*’ Indicates an interaction between two main effects.

The probability of failure increased with increasing DBH for eight species: green ash (Figure 2), red maple, white pine, pin oak, and, marginally, honeylocust, red pine, London planetree, and Liberty elm (*Ulmus americana* L. ‘American Liberty’). For red maple and Liberty elm, the correlation with DBH was only significant when a defect was not present. For green ash, evidence supporting the observation that the probability of failure increased with DBH more quickly when a defect was not present was less clear (Table 2, Figure 2). The probability of failure of littleleaf linden (*Tilia cordata* Mill.) increased when trees were in-leaf (Figure 2), unlike in red maple and Liberty elm, the only other species for which the effect of leaf was included in the logistic regression model (Table 2). The probability of failure decreased when defects were present for red maple and red oak, and, marginally, pin oak and littleleaf linden (Table 2).

### Relationships between DBH and branch morphology

Mean branch length, branch diameter and cumulative diameter of secondary branches were directly proportional to DBH, but branch slenderness was inversely proportional to DBH (Table 3). The relationships were similar for all species, except that mean branch length increased less for each unit increase in DBH for red maple, honeylocust, and littleleaf linden than green ash, red oak, Liberty elm, and London planetree (Table 3). Neither branch angle (r^2^ = 0.00) nor azimuth (r^2^ = 0.00) varied with DBH. Branch azimuth did not vary among species (p = 0.096), but branch angle was smaller for Liberty elm (mean = 38) than red maple (mean = 63, p = 0.022), honeylocust (mean = 70, p = 0.004), and London planetree (mean = 64, p = 0.017). The cumulative diameter of secondary branches was inversely and non-linearly proportional to branch slenderness for all species of deciduous trees (Table 4). The slope of the relationship was similar for primary branches of pin oak that did and did not fail (p = 0.176), but the intercept was greater (p = 0.047) for branches that failed (3.56 ± 1.77) than did not fail (0.04 ± 0.95).

**Table 3:** **Analysis of covariance tables for comparison between species including DBH as a covariate for mean slenderness, diameter, length, and cumulative diameter of secondary branches on three individuals of seven angiosperm species (listed in the text)**

		Slenderness			Diameter			Cumulative diameter			Length		
Source	DF	MS	F	p	MS	F	p	MS	F	p	MS	F	p
**Species**	6	44	1.23	0.394	5.90E-05	0.64	0.698	0.002	1.47	0.312	0.117	1.43	0.323
**DBH**	1	361	10.2	0.015	1.00E-02	113	<0.001	0.142	125	<0.001	14.8	181	<0.001
DBH*Species	6	71	2	0.193	1.60E-04	1.75	0.24	0.004	3.12	0.081	0.32	3.91	0.049
**Best-fit line**		67 – 17 × DBH			0.02 + 0.19 × DBH			0.01 + 0.59 × DBH			2.39 + 12.2 × DBH^y^		
											2.39 + 5.01 × DBH^x^		
**r** ^**2**^		0.86			0.97			0.97			0.99		

‘*’ Indicates an interaction between main effects.

Excepting branch length, one best-fit line described the relationship between DBH and each response variable for all species.

^**y**^Green ash, London planetree, red oak, Liberty elm.

^**x**^Red maple, honeylocust, littleleaf linden.

**Table 4:** **Analysis of covariance table for comparison between angiosperm species of the cumulative diameter of secondary branches including primary branch slenderness as a covariate**

Source	DF	MS	F	p
**Species**	7	4.02	0.79	0.614
**Slenderness**	1	522	102	<0.001
DBH*Slenderness	7	7.61	1.49	0.157
**Best-fit line**	1.43 - 0.30 × ln(slenderness)			
**r** ^**2**^	0.35			

‘*’ Indicates an interaction between main effects.

### Branch morphology of pin oaks

Of 495 branches on 9 pin oaks, 96 failed, but only 11.5% of those were defective. All of the defective branches were weakly attached. Excluding branches that failed at a defect, the only morphologic differences between branches that did and did not fail was that the cumulative diameter and length of higher-order branches on branches that failed was significantly greater than branches that did not fail (Table 5). No other morphologic differences existed between branches that did and did not fail (Table 5).

**Table 5:** **Comparison of attributes of branches that failed or did not fail for nine pin oaks**

Parameter	Not fail	n	Fail	n	p
**Height (m)**	7.97 (0.58)	117	9.04 (0.69)	47	0.314
**Diameter (cm)**	15.3 (1.22)	117	16.4 (1.37)	47	0.416
**Azimuth (°)**	182 (5.38)	117	191 (10.49)	47	0.809
**Angle (°)**	49.5 (4.69)	117	44.5 (5.25)	47	0.443
**Length (m)**	4.69 (0.18)	392	4.90 (0.22)	93	0.352
**Slenderness** ^**z**^ **(m/m)**	1.67 (0.10)	388	1.61 (0.15)	91	0.214
Σ**(Diameter)**^**y**^**(cm)**	4.90 (0.70)	398	9.73 (1.45)	95	0.022
Σ**(Length)**^**x**^**(cm)**	2.47 (0.30)	398	5.49(0.68)	95	<0.001

Means are followed by standard errors in parentheses.

^**z**^length/diameter.

^**y**^Cumulative diameter of lateral branches.

^**x**^Cumulative length of lateral branches.

## Discussion

The few stem and root failures was not surprising given the static nature of the snow load, low wind speed, and the decurrent form of most trees. Subjected to snow loads, forest-grown conifers (Nykänen et al. 1997) and leafless deciduous trees (Zhu et al. 2006) with less slender stems have been more prone to crown breakage than stem breakage, as we also observed. We also expected that both pine species would experience relatively minor damage because of their ability to shed snow. Pines sometimes suffer greater damage than deciduous trees during ice storms (Warrillow and Mou 1999), because ice cannot be shed as readily. When leafless, deciduous trees are often less likely to suffer snow-induced failure than evergreens (Nykänen et al. 1997; Peltola et al. 1997; Päätalo et al. 1999; Päätalo 2000).

The aberrant timing of the snowstorm may explain inconsistencies with previous work in urbanized settings. For example, red oak and littleleaf linden have previously been considered resistant to ice damage (Hauer et al. 1993; Sisinni et al. 1995), but had a high probability of snow-induced failure. The presence of leaves presumably facilitated the accumulation of snow on branches. Conversely, green ash has been considered susceptible to ice damage (Hauer et al. 1993; Sisinni et al. 1995), but the absence of leaves on trees in the current study reduced the probability of snow-induced failure.

We expected that the probability of failure would be greater for larger trees for three synergistic reasons. First, leaf area increases non-linearly with branch diameter for many species (Cummings 1941; Rothacher et al. 1954; Weiskittel et al. 2009), and both primary branch diameter and the cumulative diameter of secondary branches of seven angiosperm species increased in proportion to DBH. Leaves (and needles) obviously provide greater surface area on which snow accumulates (Petty and Worrell 1981; Peltola et al. 1999), and primary branches with secondary branches accumulate more snow than those without (Cannell and Morgan 1989). However, differences in leaf area were not consistent with the finding that the rate of failure of both *Pinus* species, individuals of which were all in leaf, was less than or similar to the rate of failure of the two angiosperms with the fewest in-leaf individuals (green ash and honeylocust). The ability to shed snow is also important, and the second reason that trees with greater DBH were more likely to fail is that their larger diameter branches were less flexible and less able to shed snow (Cannell and Morgan 1989). Since primary branch slenderness decreased with increasing DBH for seven angiosperm species, flexibility was further diminished on larger trees. Less slender primary branches also had greater cumulative diameter of secondary branches, so the branches least able to shed snow also were more likely to accumulate snow. Of the two factors, the latter may have been more important for deciduous trees that retained their leaves because pin oak branches that failed had greater cumulative diameter of secondary branches, but this was not true of slenderness. Thirdly, both the increase in primary branch length of seven angiosperm species with greater DBH and the greater cumulative length of higher-order branches on failed pin oak branches mean that the snow-induced bending moment on branches would have increased. Previous observations of forest-grown (Van Dyke 1999; Proulx and Greene 2001) and open-grown (Hauer et al. 1993; Sisinni et al. 1995; Rhoades and Stipes 2007) trees subjected to ice loads have also shown that the probability of branch failure increased with tree size. This is mechanically intuitive because bending stress decreases with the cube of diameter. Although Nock et al. (2013) showed that ice accreted more on branches higher in the crown, we did not observe any differences in the height of pin oak branches that did and did not fail.

For most deciduous species, there were too few individuals to test the effect of leaf with logistic regression. However, the presence (London planetree, pin oak and red oak) or absence (green ash and honeylocust) of leaves presumably contributed to the probability of failure, which was in general greater for species in which most individuals were in-leaf. However, when we included the effect of leaf in logistic regression models (red maple, littleleaf linden, Liberty elm), probability of failure was correlated with the presence of leaves only for littleleaf linden. This disparity may have been due to characterizing trees rather than individual branches that failed as in-leaf or leafless. Although the percentages of red maples and Liberty elms that failed and failed at a defect were similar, as was mean DBH for both species, the probability of failure was noticeably greater for Liberty elms. Although a greater percentage of red maples had leaves, the cumulative diameter of secondary branches for Liberty elms was two and a half times greater than that for red maples. According to Nowak (1996), leaf area for a certain DBH for American elm is greater than red maple.

Defects like weakly attached branches played a minor role in failures recorded during the storm. This was evident in the small percentage of failed branches associated with a defect on all trees, as well as the nine intensively-measured pin oaks. It was also evident in the counter-intuitive finding for four species that the presence of a defect reduced the probability of failure. Two factors may explain this finding. First, we did not assess the severity of defects, a clear limitation of our work. By convention (Smiley et al. 2011), we considered weakly attached branches as those with a narrow attachment, included bark, visually similar diameters of branch and trunk, or a combination of these. Secondly, for most structural defects of trees, thresholds at which failure is more likely have not been established [see, for example, Kane (2014)]. Regarding the most common defect we observed (weakly attached branches), previous studies have suggested that the strength of a branch attachment decreases (i) as the ratio of the branch and trunk diameters increases (Kane et al. 2008) and (ii) with the presence of included bark (Smiley 2003), and these findings have been consistent among several species (Kane et al. 2008). Only recently, however, has a more detailed investigation of the mechanism of the failure of branch attachments (Slater and Ennos 2013) been undertaken.

Aside from differences between angiosperms and gymnosperms or the timing of leaf senescence for different species, tree size and its influence of branch morphology mostly superseded the effect of species. The only species differences were of attachment angle and the proportionally greater increase in branch length with DBH for green ash, London planetree, red oak, and Liberty elm, compared to red maple, honeylocust, and littleleaf linden. In combination with the presence of leaves on most London planetrees and red oaks, greater branch length would increase snow-induced bending moments on branches of those species. We observed many weakly attached branches on Liberty elms, which is consistent with the smaller mean attachment angles measured on that species, as well as the number of defective branches that did not fail. Attachment angle may affect snow accumulation, and can also be associated with the presence of included bark, which we recorded as a defect and reduces the strength of branch attachments (Smiley 2003). MOR also differs among species (Kretschmann 2010), but the lack of correlation between MOR and the probability of failure was not surprising considering that failure depends on both applied stress and MOR: stronger wood only reduces the likelihood of failure if the snow-induced bending stress is similar between two branches.

It is expected that the frequency of intense storms will increase in the future, increasing the likelihood of wind- and snow-induced damage to trees growing in urbanized settings. Practitioners should consider such risks in planning the urban forest and in managing individual trees. There are many reasons to plant a diversity of species in urban landscapes. Our results suggest that urban foresters should also consider the timing of leaf senescence when selecting deciduous trees, to reduce the likelihood of failure in temperate climates. Pruning can also alter the likelihood of failure. Reduction pruning—shortening branches by removing the distal portion of a parent branch back to a higher-order branch (Gilman and Lilly 2008)—can be used to reduce drag-induced bending moment (Smiley and Kane 2006; Pavlis et al. 2008) and increase natural frequency (Kane and James 2011). Prior to re-growth following pruning, shortening branch length also reduces the snow-induced bending moment, but it may inhibit snow shedding because the shorter branch is less slender and more stiff . In contrast, thinning—removing higher order branches from a primary branch (Gilman and Lilly 2008)—does not, in the short-term, alter branch slenderness, but does reduce the cumulative diameter of secondary branches and the leaf area on which snow can accumulate. Practitioners should consider the likelihood of different types of loading with respect to leaf senescence when deciding which type of pruning may be most effective to reduce the likelihood of failure.

## Conclusions

The main driver of branch failure in our work appeared to be accumulation of snow, expedited by the presence of leaves and the inability of larger, less flexible branches to shed it. However, our observations are limited by cursory measurements of defects and leaves on trees observed immediately after the snowstorm. We justify this approach given our objectives (to explore general trends among species—our work is the first to examine snow-induced failure of open-grown trees in urbanized areas), and because time constraints required that multiple observers collected data prior to removal of damaged branches. Our results provide a useful foundation for subsequent work to investigate in greater detail snow- and ice-induced branch failures of open-grown trees in urbanized areas. A better understanding of this will help guide management practices like pruning to reduce the likelihood of snow-induced branch failure.

### Key message

The likelihood of branch failure increased as diameter at breast height increased, and differed among species commonly planted in urban areas in northeastern USA.

## Authors’ original submitted files for images

Below are the links to the authors’ original submitted files for images. Authors’ original file for figure 1 Authors’ original file for figure 2
