# Supplementary figures and images for: Factors affecting branch failures in open-grown trees during a snowstorm in Massachusetts, USA
Source: Springerplus. 2014 Dec 10;3:720. doi: 10.1186/2193-1801-3-720 (PMC4320161; doi:10.1186/2193-1801-3-720)

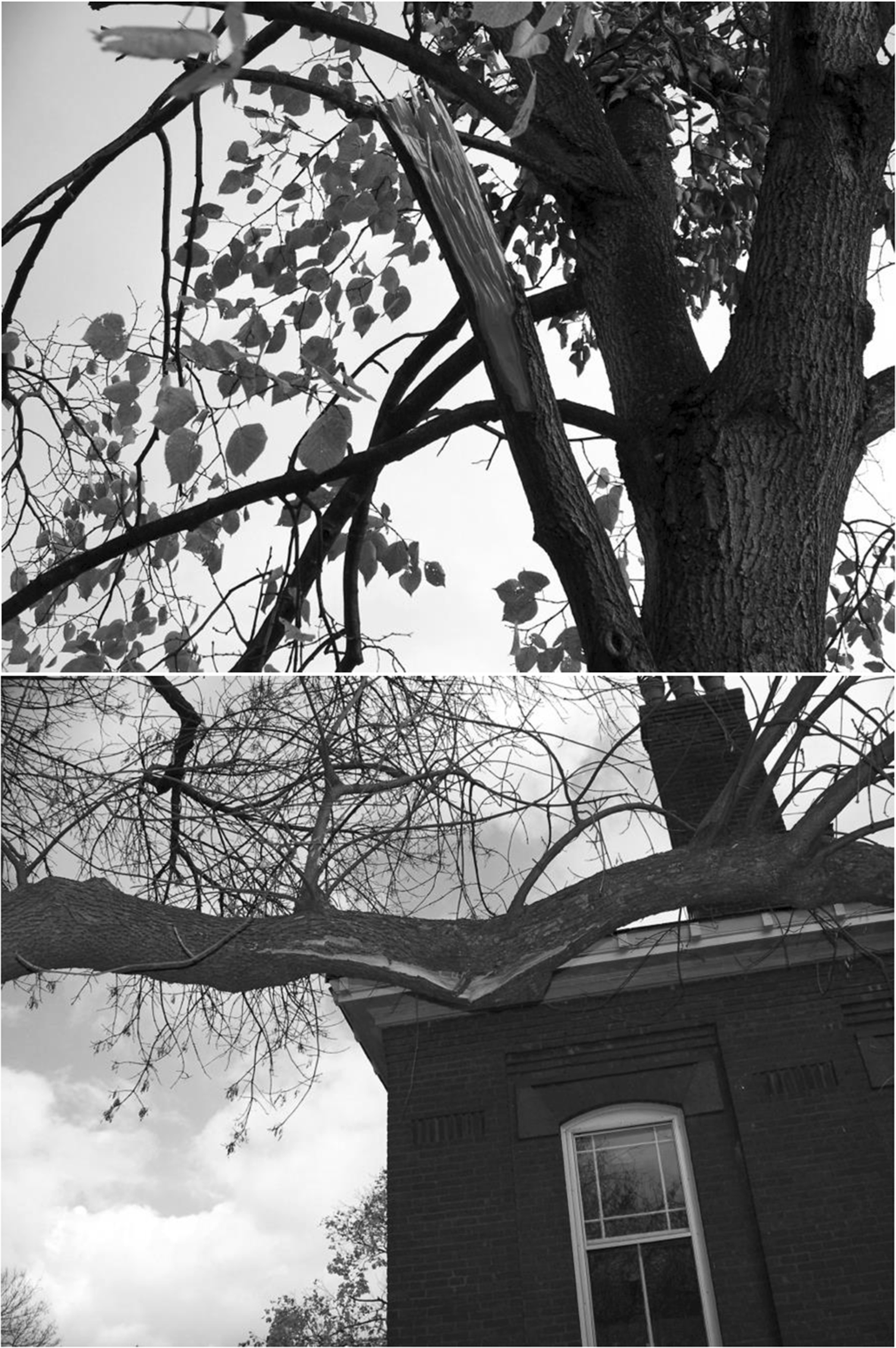

Supplement: Supplementary file 1 — Authors’ original file for figure 1 [file 40064_2014_1494_MOESM1_ESM.tif]

**a**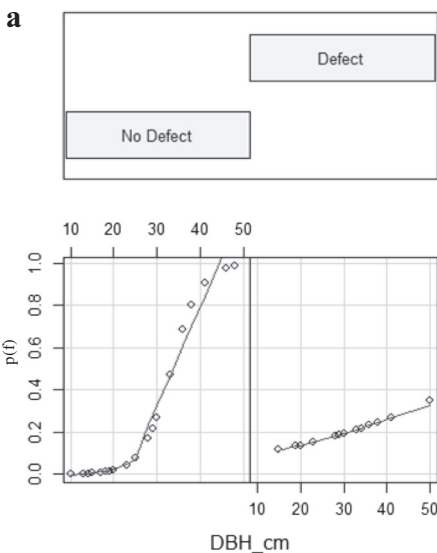**b**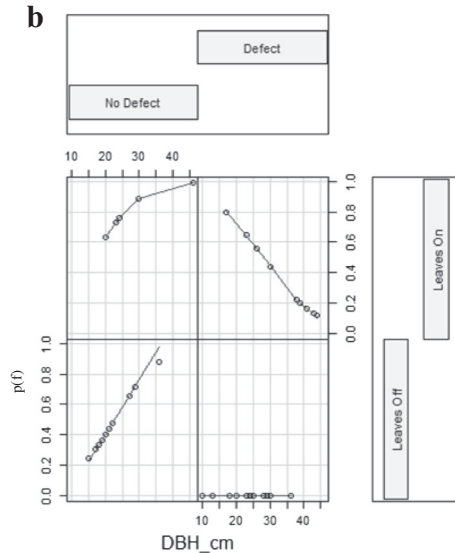**c**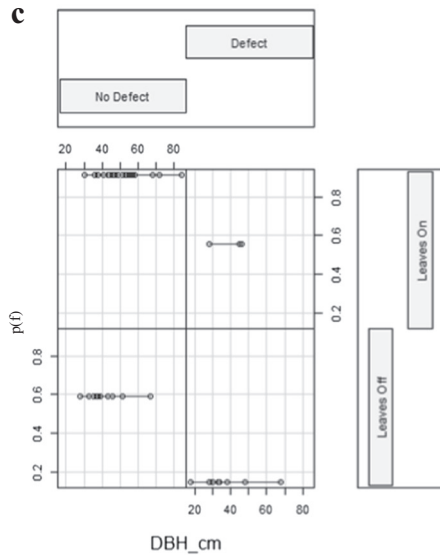

Supplement: Supplementary file 2 — Authors’ original file for figure 2 [file 40064_2014_1494_MOESM2_ESM.pdf]
